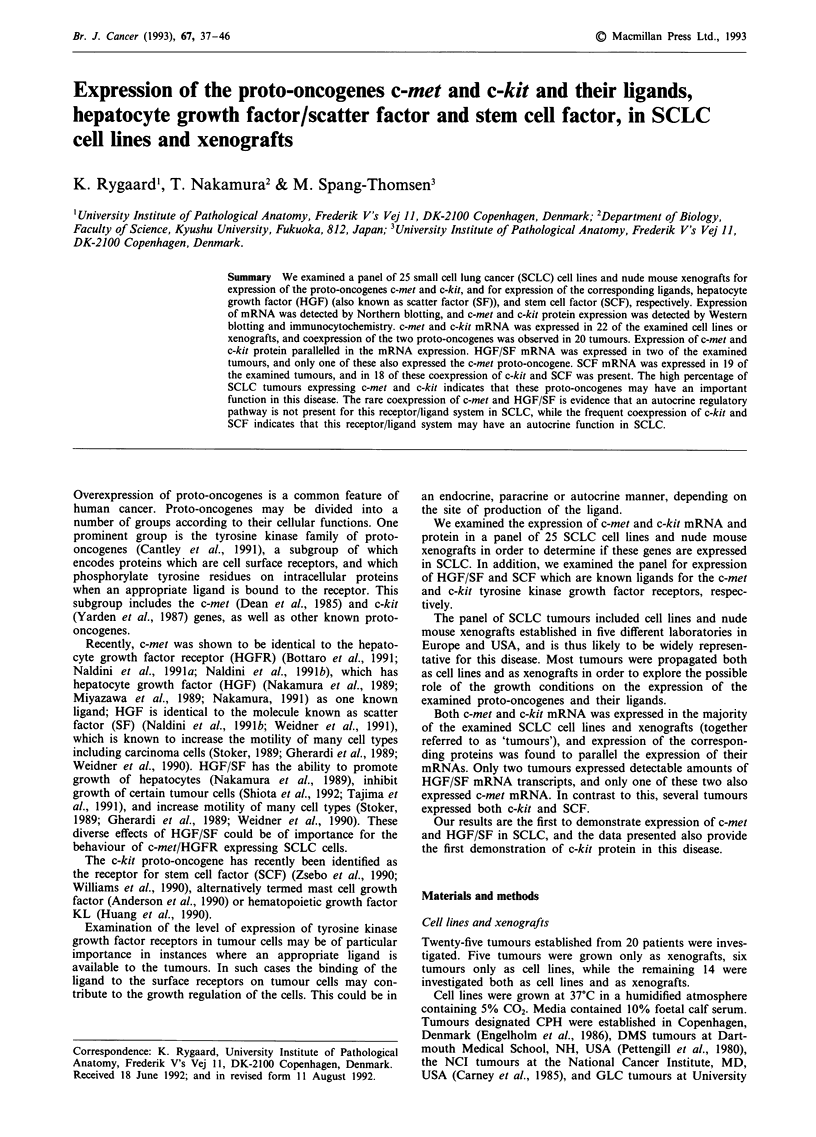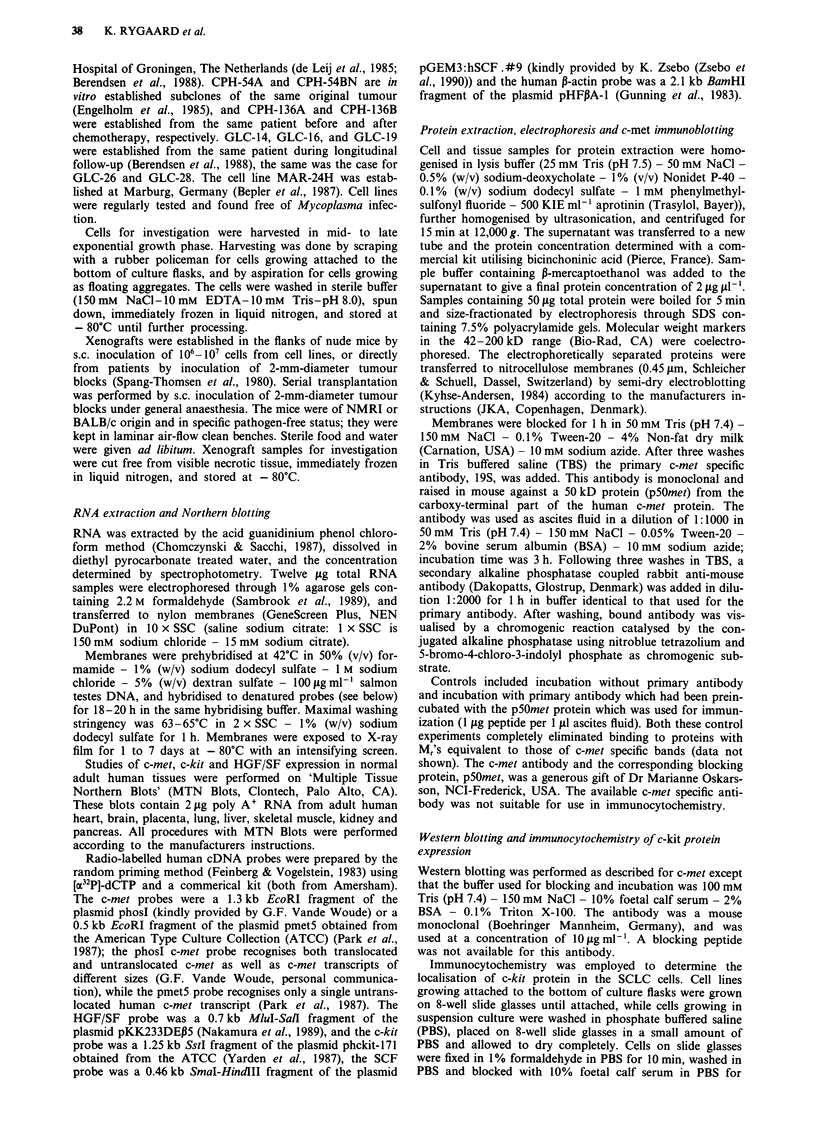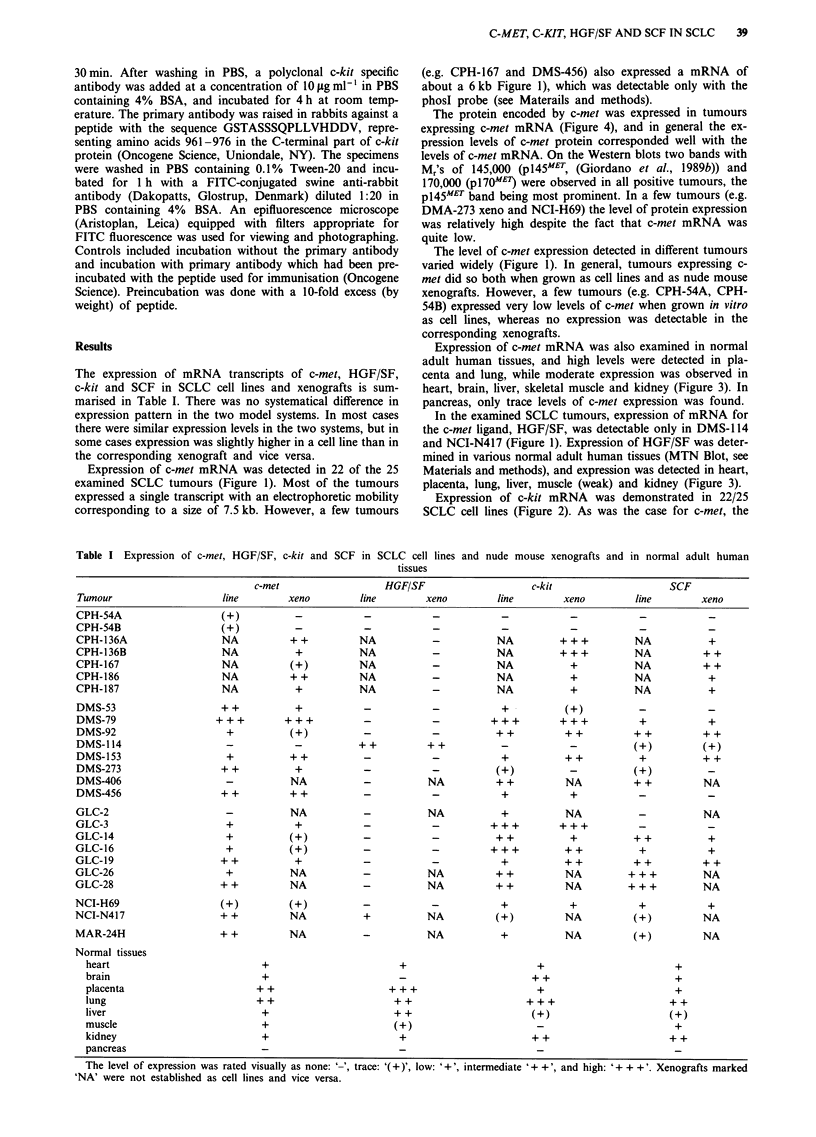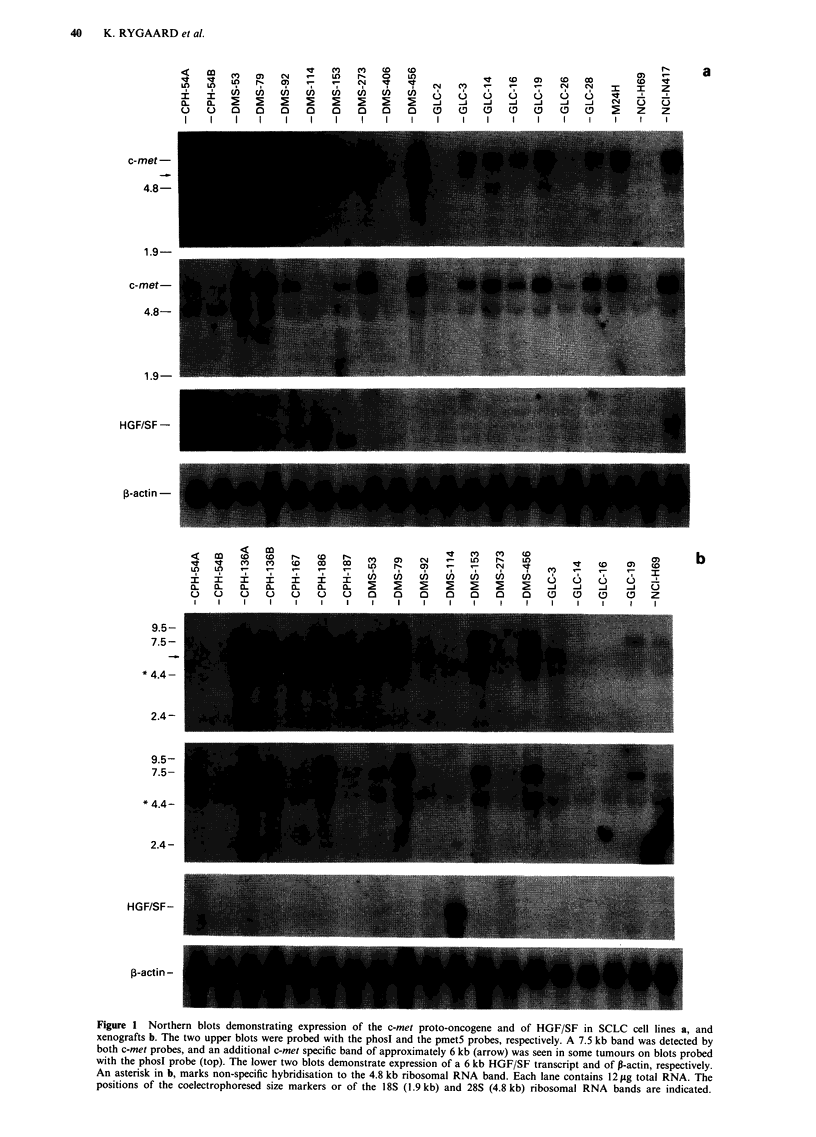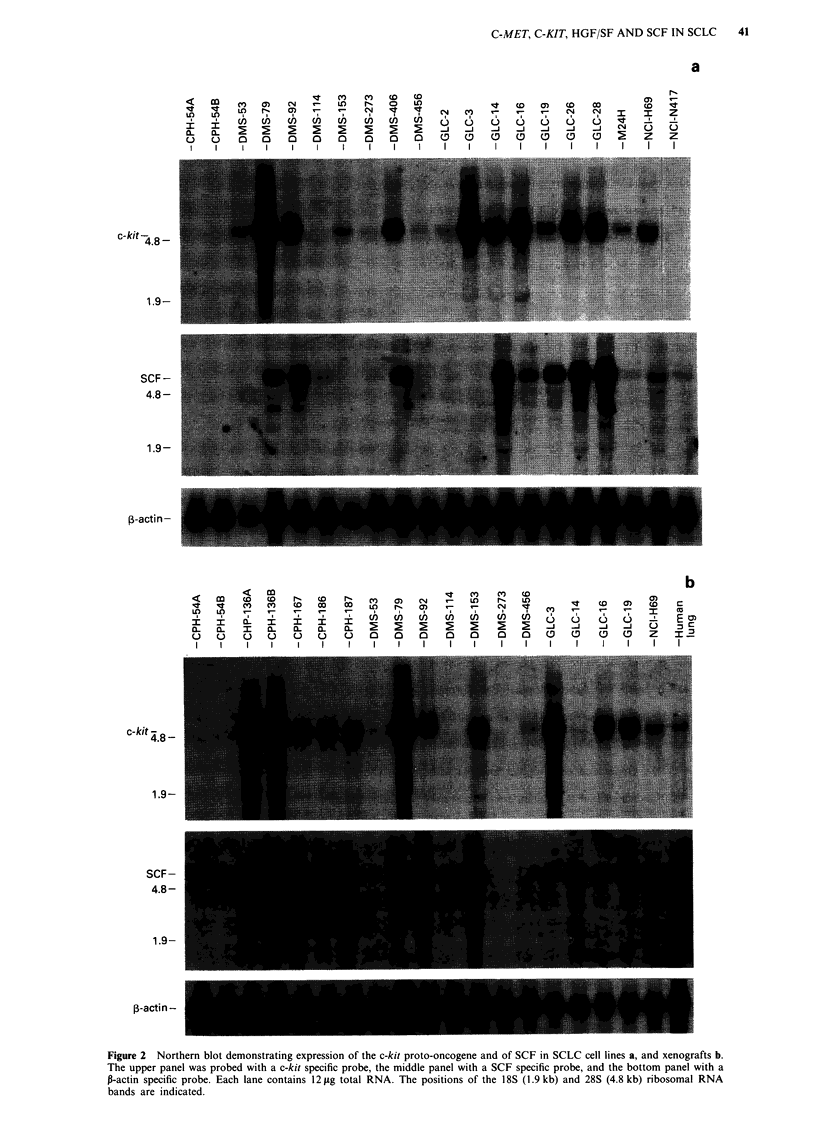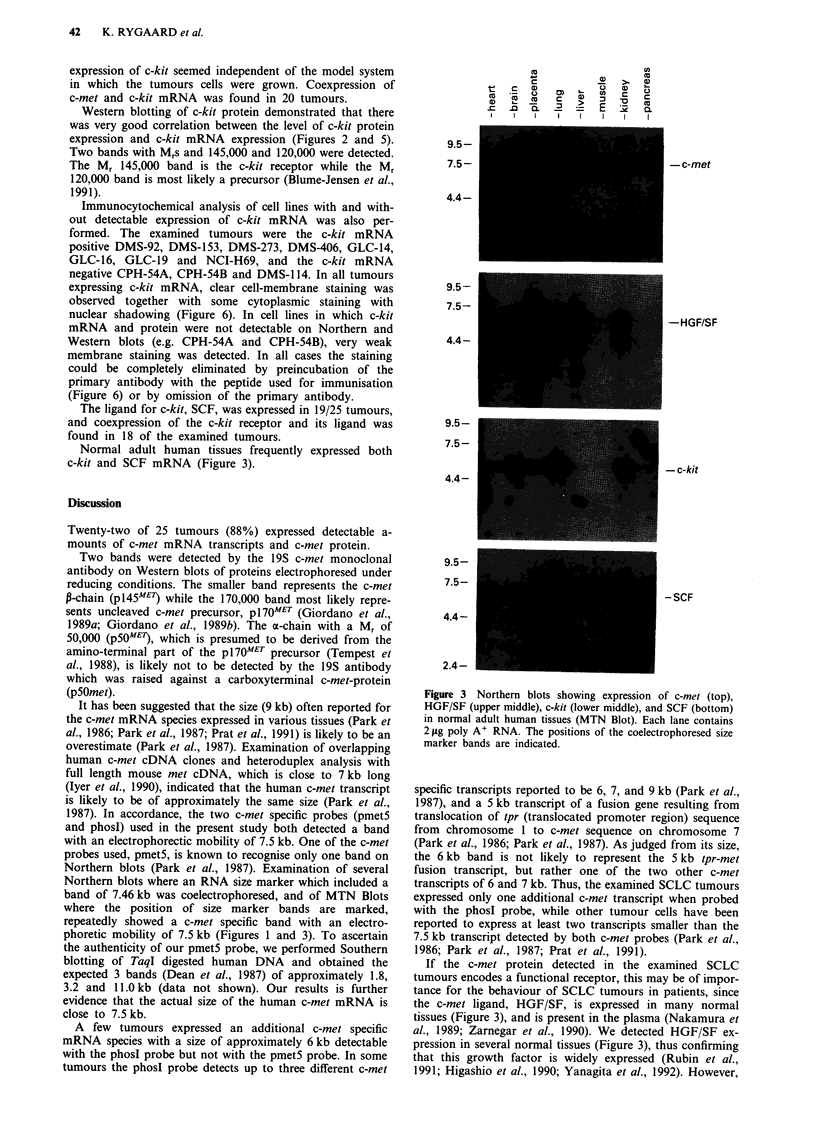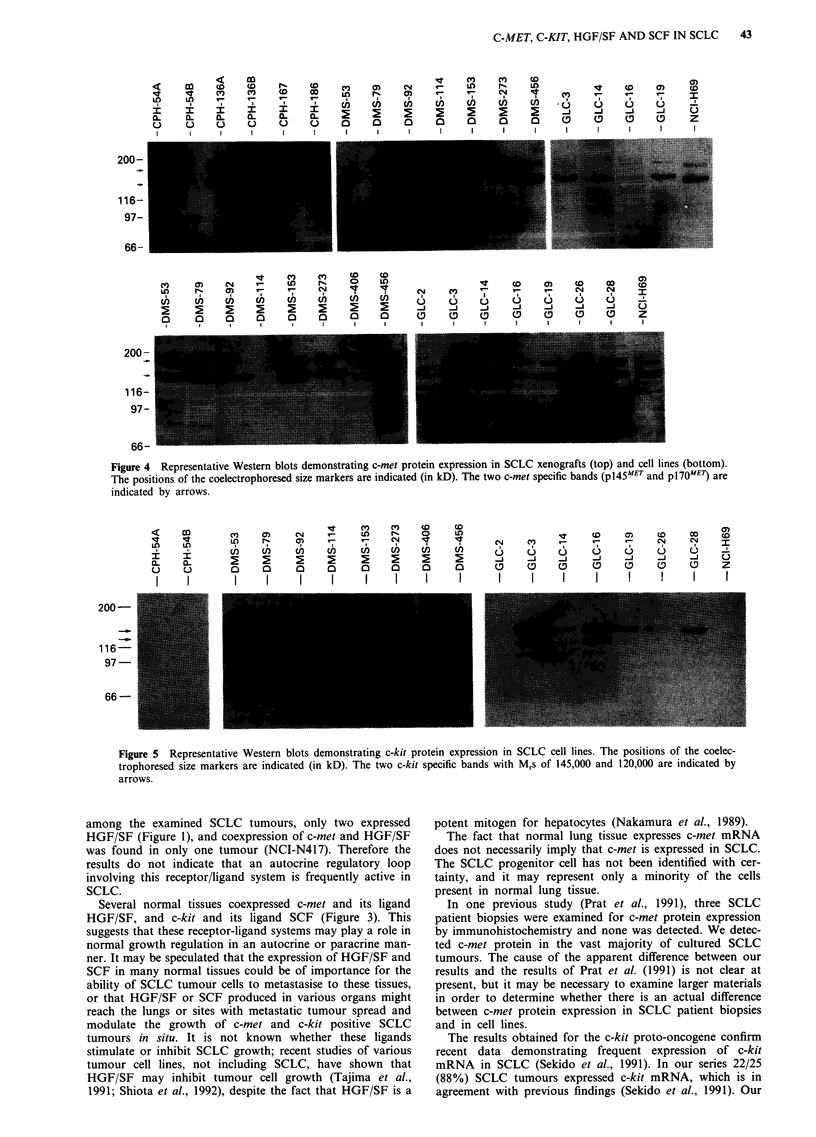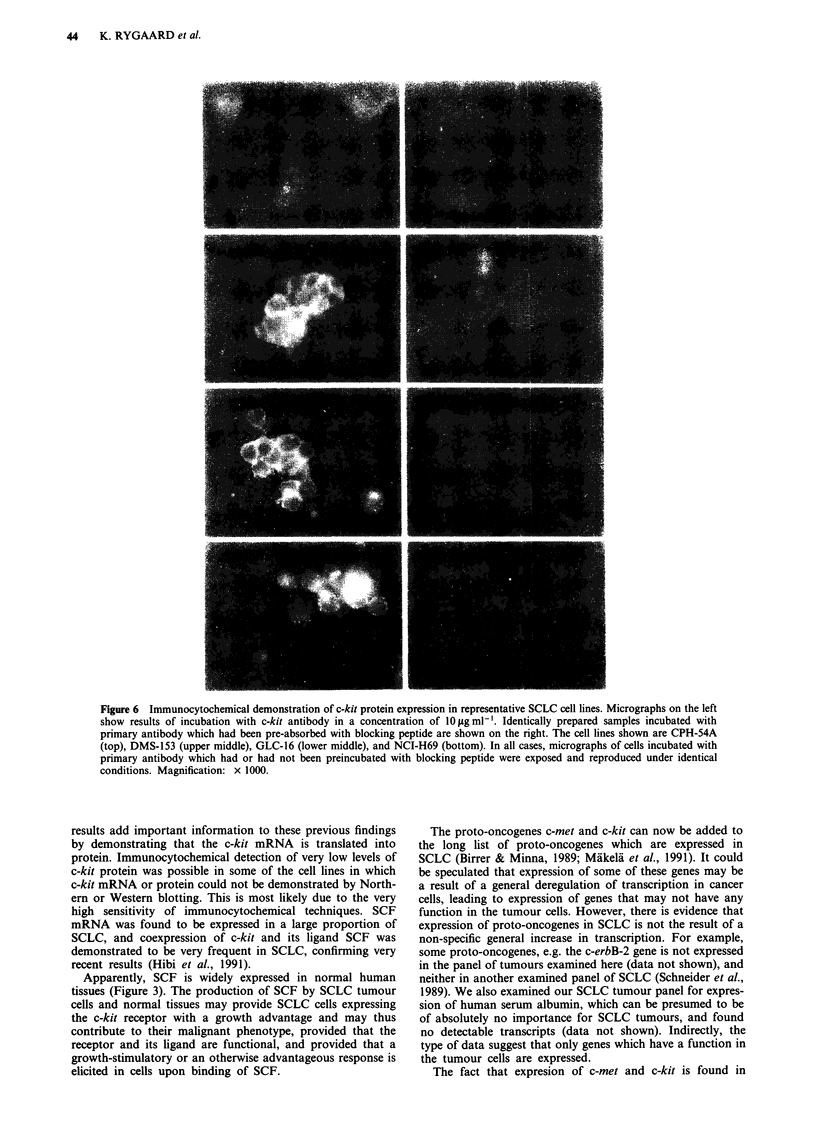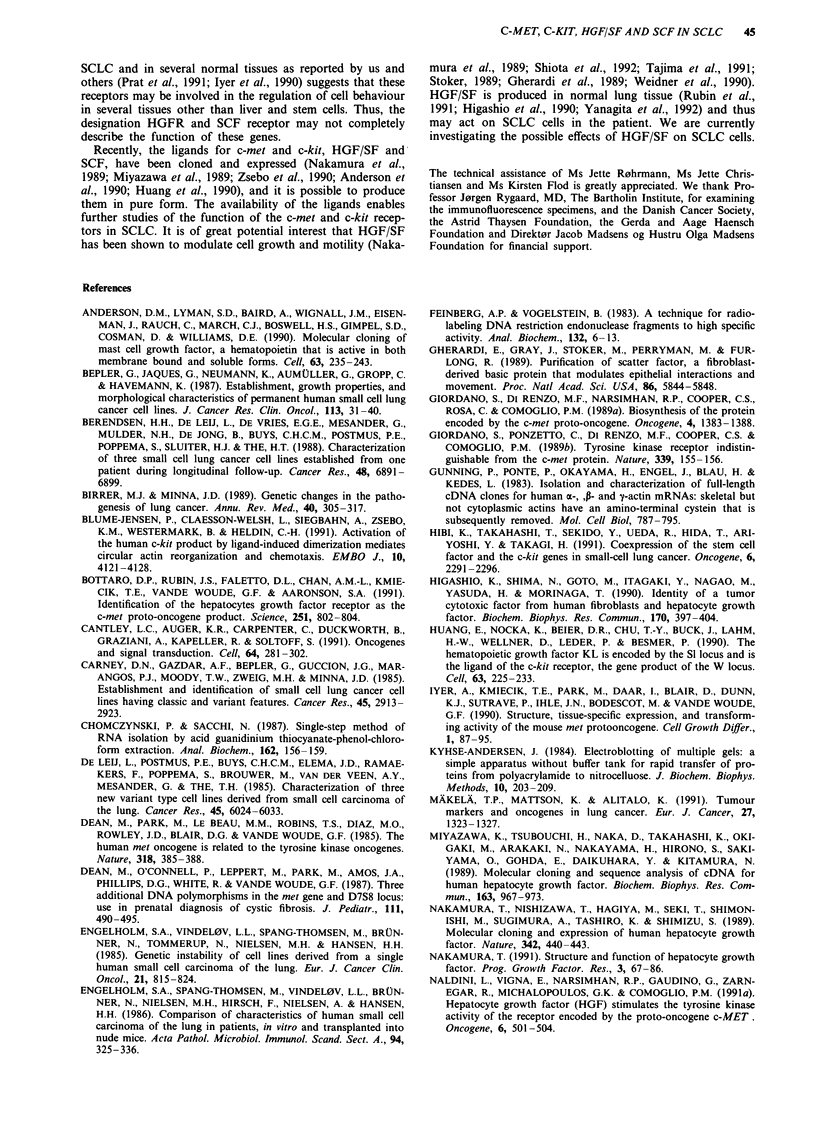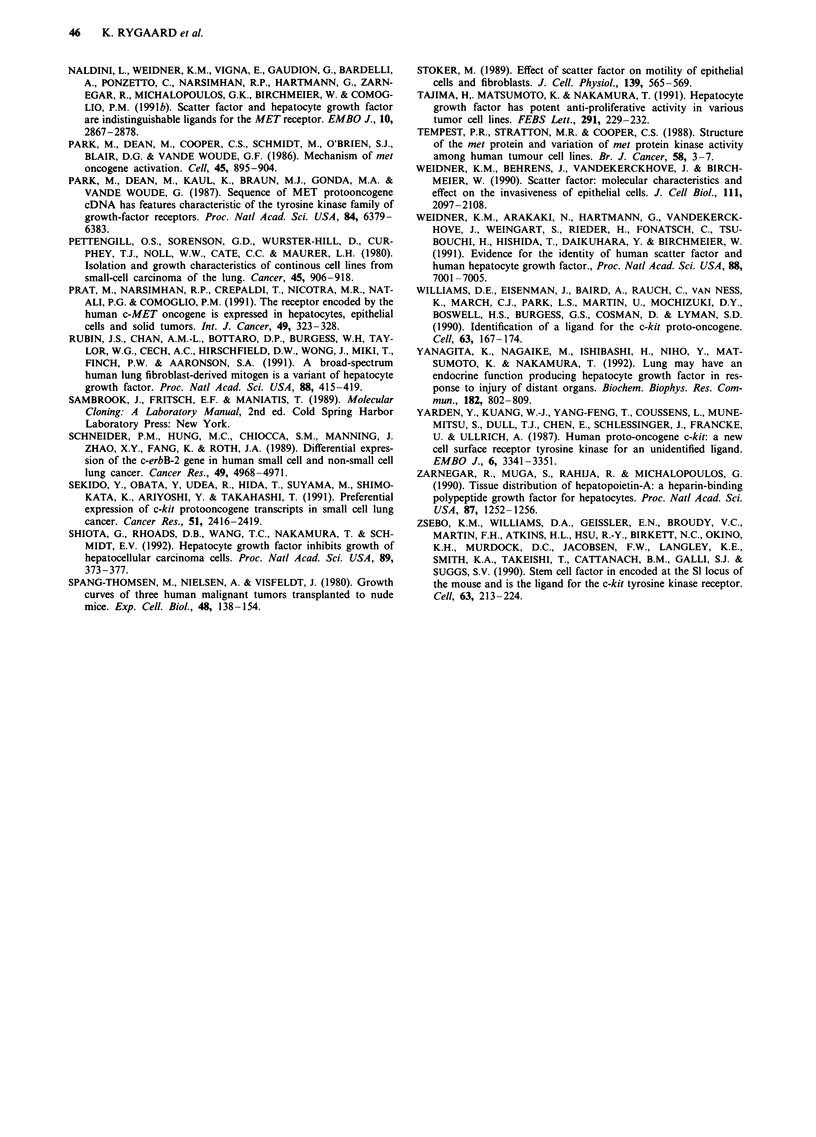# Expression of the proto-oncogenes c-met and c-kit and their ligands, hepatocyte growth factor/scatter factor and stem cell factor, in SCLC cell lines and xenografts.

**DOI:** 10.1038/bjc.1993.7

**Published:** 1993-01

**Authors:** K. Rygaard, T. Nakamura, M. Spang-Thomsen

**Affiliations:** University Institute of Pathological Anatomy, Copenhagen, Denmark.

## Abstract

**Images:**


Br. J. Cancer (1993), 67, 37-46 © Macmillan Press Ltd., 1993

### Expression of the proto-oncogenes c-*met* and c-*kit* and their ligands, hepatocyte growth factor/scatter factor and stem cell factor, in SCLC cell lines and xenografts

K. Rygaard[1], T. Nakamura[2] & M. Spang-Thomsen[3]

[1]*University Institute of Pathological Anatomy, Frederik V's Vej 11, DK-2100 Copenhagen, Denmark;* [2]*Department of Biology, Faculty of Science, Kyushu University, Fukuoka, 812, Japan;* [3]*University Institute of Pathological Anatomy, Frederik V's Vej 11, DK-2100 Copenhagen, Denmark.*

**Summary** We examined a panel of 25 small cell lung cancer (SCLC) cell lines and nude mouse xenografts for expression of the proto-oncogenes c-*met* and c-*kit*, and for expression of the corresponding ligands, hepatocyte growth factor (HGF) (also known as scatter factor (SF)), and stem cell factor (SCF), respectively. Expression of mRNA was detected by Northern blotting, and c-*met* and c-*kit* protein expression was detected by Western blotting and immunocytochemistry. c-*met* and c-*kit* mRNA was expressed in 22 of the examined cell lines or xenografts, and coexpression of the two proto-oncogenes was observed in 20 tumours. Expression of c-*met* and c-*kit* protein parallelled in the mRNA expression. HGF/SF mRNA was expressed in two of the examined tumours, and only one of these also expressed the c-*met* proto-oncogene. SCF mRNA was expressed in 19 of the examined tumours, and in 18 of these coexpression of c-*kit* and SCF was present. The high percentage of SCLC tumours expressing c-*met* and c-*kit* indicates that these proto-oncogenes may have an important function in this disease. The rare coexpression of c-*met* and HGF/SF is evidence that an autocrine regulatory pathway is not present for this receptor/ligand system in SCLC, while the frequent coexpression of c-*kit* and SCF indicates that this receptor/ligand system may have an autocrine function in SCLC.

Overexpression of proto-oncogenes is a common feature of human cancer. Proto-oncogenes may be divided into a number of groups according to their cellular functions. One prominent group is the tyrosine kinase family of proto-oncogenes (Cantley *et al.*, 1991), a subgroup of which encodes proteins which are cell surface receptors, and which phosphorylate tyrosine residues on intracellular proteins when an appropriate ligand is bound to the receptor. This subgroup includes the c-*met* (Dean *et al.*, 1985) and c-*kit* (Yarden *et al.*, 1987) genes, as well as other known proto-oncogenes.

Recently, c-*met* was shown to be identical to the hepatocyte growth factor receptor (HGFR) (Bottaro *et al.*, 1991; Naldini *et al.*, 1991*a*; Naldini *et al.*, 1991*b*), which has hepatocyte growth factor (HGF) (Nakamura *et al.*, 1989; Miyazawa *et al.*, 1989; Nakamura, 1991) as one known ligand; HGF is identical to the molecule known as scatter factor (SF) (Naldini *et al.*, 1991*b*; Weidner *et al.*, 1991), which is known to increase the motility of many cell types including carcinoma cells (Stoker, 1989; Gherardi *et al.*, 1989; Weidner *et al.*, 1990). HGF/SF has the ability to promote growth of hepatocytes (Nakamura *et al.*, 1989), inhibit growth of certain tumour cells (Shiota *et al.*, 1992; Tajima *et al.*, 1991), and increase motility of many cell types (Stoker, 1989; Gherardi *et al.*, 1989; Weidner *et al.*, 1990). These diverse effects of HGF/SF could be of importance for the behaviour of c-*met*/HGFR expressing SCLC cells.

The c-*kit* proto-oncogene has recently been identified as the receptor for stem cell factor (SCF) (Zsebo *et al.*, 1990; Williams *et al.*, 1990), alternatively termed mast cell growth factor (Anderson *et al.*, 1990) or hematopoietic growth factor KL (Huang *et al.*, 1990).

Examination of the level of expression of tyrosine kinase growth factor receptors in tumour cells may be of particular importance in instances where an appropriate ligand is available to the tumours. In such cases the binding of the ligand to the surface receptors on tumour cells may contribute to the growth regulation of the cells. This could be in an endocrine, paracrine or autocrine manner, depending on the site of production of the ligand.

We examined the expression of c-*met* and c-*kit* mRNA and protein in a panel of 25 SCLC cell lines and nude mouse xenografts in order to determine if these genes are expressed in SCLC. In addition, we examined the panel for expression of HGF/SF and SCF which are known ligands for the c-*met* and c-*kit* tyrosine kinase growth factor receptors, respectively.

The panel of SCLC tumours included cell lines and nude mouse xenografts established in five different laboratories in Europe and USA, and is thus likely to be widely representative for this disease. Most tumours were propagated both as cell lines and as xenografts in order to explore the possible role of the growth conditions on the expression of the examined proto-oncogenes and their ligands.

Both c-*met* and c-*kit* mRNA was expressed in the majority of the examined SCLC cell lines and xenografts (together referred to as 'tumours'), and expression of the corresponding proteins was found to parallel the expression of their mRNAs. Only two tumours expressed detectable amounts of HGF/SF mRNA transcripts, and only one of these two also expressed c-*met* mRNA. In contrast to this, several tumours expressed both c-*kit* and SCF.

Our results are the first to demonstrate expression of c-*met* and HGF/SF in SCLC, and the data presented also provide the first demonstration of c-*kit* protein in this disease.

### Materials and methods

### *Cell lines and xenografts*

Twenty-five tumours established from 20 patients were investigated. Five tumours were grown only as xenografts, six tumours only as cell lines, while the remaining 14 were investigated both as cell lines and as xenografts.

Cell lines were grown at 37°C in a humidified atmosphere containing 5% $CO_2$. Media contained 10% foetal calf serum. Tumours designated CPH were established in Copenhagen, Denmark (Engelholm *et al.*, 1986), DMS tumours at Dartmouth Medical School, NH, USA (Pettengill *et al.*, 1980), the NCI tumours at the National Cancer Institute, MD, USA (Carney *et al.*, 1985), and GLC tumours at University

Correspondence: K. Rygaard, University Institute of Pathological Anatomy, Frederik V's Vej 11, DK-2100 Copenhagen, Denmark.
Received 18 June 1992; and in revised form 11 August 1992.

Hospital of Groningen, The Netherlands (de Leij et al., 1985; Berendsen et al., 1988). CPH-54A and CPH-54BN are *in vitro* established subclones of the same original tumour (Engelholm et al., 1985), and CPH-136A and CPH-136B were established from the same patient before and after chemotherapy, respectively. GLC-14, GLC-16, and GLC-19 were established from the same patient during longitudinal follow-up (Berendsen et al., 1988), the same was the case for GLC-26 and GLC-28. The cell line MAR-24H was established at Marburg, Germany (Bepler et al., 1987). Cell lines were regularly tested and found free of *Mycoplasma* infection.

Cells for investigation were harvested in mid- to late exponential growth phase. Harvesting was done by scraping with a rubber policeman for cells growing attached to the bottom of culture flasks, and by aspiration for cells growing as floating aggregates. The cells were washed in sterile buffer (150 mM NaCl–10 mM EDTA–10 mM Tris–pH 8.0), spun down, immediately frozen in liquid nitrogen, and stored at − 80°C until further processing.

Xenografts were established in the flanks of nude mice by s.c. inoculation of $10^6$–$10^7$ cells from cell lines, or directly from patients by inoculation of 2-mm-diameter tumour blocks (Spang-Thomsen et al., 1980). Serial transplantation was performed by s.c. inoculation of 2-mm-diameter tumour blocks under general anaesthesia. The mice were of NMRI or BALB/c origin and in specific pathogen-free status; they were kept in laminar air-flow clean benches. Sterile food and water were given *ad libitum*. Xenograft samples for investigation were cut free from visible necrotic tissue, immediately frozen in liquid nitrogen, and stored at − 80°C.

*RNA extraction and Northern blotting*

RNA was extracted by the acid guanidinium phenol chloroform method (Chomczynski & Sacchi, 1987), dissolved in diethyl pyrocarbonate treated water, and the concentration determined by spectrophotometry. Twelve μg total RNA samples were electrophoresed through 1% agarose gels containing 2.2 M formaldehyde (Sambrook et al., 1989), and transferred to nylon membranes (GeneScreen Plus, NEN DuPont) in 10 × SSC (saline sodium citrate: 1 × SSC is 150 mM sodium chloride – 15 mM sodium citrate).

Membranes were prehybridised at 42°C in 50% (v/v) formamide – 1% (w/v) sodium dodecyl sulfate – 1 M sodium chloride – 5% (w/v) dextran sulfate – 100 μg $ml^{-1}$ salmon testes DNA, and hybridised to denatured probes (see below) for 18–20 h in the same hybridising buffer. Maximal washing stringency was 63–65°C in 2 × SSC – 1% (w/v) sodium dodecyl sulfate for 1 h. Membranes were exposed to X-ray film for 1 to 7 days at − 80°C with an intensifying screen.

Studies of c-*met*, c-*kit* and HGF/SF expression in normal adult human tissues were performed on 'Multiple Tissue Northern Blots' (MTN Blots, Clontech, Palo Alto, CA). These blots contain 2 μg poly $A^+$ RNA from adult human heart, brain, placenta, lung, liver, skeletal muscle, kidney and pancreas. All procedures with MTN Blots were performed according to the manufacturers instructions.

Radio-labelled human cDNA probes were prepared by the random priming method (Feinberg & Vogelstein, 1983) using [$\alpha^{32}$P]-dCTP and a commerical kit (both from Amersham). The c-*met* probes were a 1.3 kb *Eco*RI fragment of the plasmid phosI (kindly provided by G.F. Vande Woude) or a 0.5 kb *Eco*RI fragment of the plasmid pmet5 obtained from the American Type Culture Collection (ATCC) (Park et al., 1987); the phosI c-*met* probe recognises both translocated and untranslocated c-*met* as well as c-*met* transcripts of different sizes (G.F. Vande Woude, personal communication), while the pmet5 probe recognises only a single untranslocated human c-*met* transcript (Park et al., 1987). The HGF/SF probe was a 0.7 kb *Mlu*I-*Sal*I fragment of the plasmid pKK233DEβ5 (Nakamura et al., 1989), and the c-*kit* probe was a 1.25 kb *Sst*I fragment of the plasmid phckit-171 obtained from the ATCC (Yarden et al., 1987), the SCF probe was a 0.46 kb *Sma*I-*Hind*III fragment of the plasmid pGEM3:hSCF.#9 (kindly provided by K. Zsebo (Zsebo et al., 1990)) and the human β-actin probe was a 2.1 kb *Bam*HI fragment of the plasmid pHFβA-1 (Gunning et al., 1983).

*Protein extraction, electrophoresis and* c-met *immunoblotting*

Cell and tissue samples for protein extraction were homogenised in lysis buffer (25 mM Tris (pH 7.5) – 50 mM NaCl – 0.5% (w/v) sodium-deoxycholate – 1% (v/v) Nonidet P-40 – 0.1% (w/v) sodium dodecyl sulfate – 1 mM phenylmethylsulfonyl fluoride – 500 KIE $ml^{-1}$ aprotinin (Trasylol, Bayer)), further homogenised by ultrasonication, and centrifuged for 15 min at 12,000 *g*. The supernatant was transferred to a new tube and the protein concentration determined with a commercial kit utilising bicinchoninic acid (Pierce, France). Sample buffer containing β-mercaptoethanol was added to the supernatant to give a final protein concentration of 2 μg $\mu l^{-1}$. Samples containing 50 μg total protein were boiled for 5 min and size-fractionated by electrophoresis through SDS containing 7.5% polyacrylamide gels. Molecular weight markers in the 42–200 kD range (Bio-Rad, CA) were coelectrophoresed. The electrophoretically separated proteins were transferred to nitrocellulose membranes (0.45 μm, Schleicher & Schuell, Dassel, Switzerland) by semi-dry electroblotting (Kyhse-Andersen, 1984) according to the manufacturers instructions (JKA, Copenhagen, Denmark).

Membranes were blocked for 1 h in 50 mM Tris (pH 7.4) – 150 mM NaCl – 0.1% Tween-20 – 4% Non-fat dry milk (Carnation, USA) – 10 mM sodium azide. After three washes in Tris buffered saline (TBS) the primary c-*met* specific antibody, 19S, was added. This antibody is monoclonal and raised in mouse against a 50 kD protein (p50*met*) from the carboxy-terminal part of the human c-*met* protein. The antibody was used as ascites fluid in a dilution of 1:1000 in 50 mM Tris (pH 7.4) – 150 mM NaCl – 0.05% Tween-20 – 2% bovine serum albumin (BSA) – 10 mM sodium azide; incubation time was 3 h. Following three washes in TBS, a secondary alkaline phosphatase coupled rabbit anti-mouse antibody (Dakopatts, Glostrup, Denmark) was added in dilution 1:2000 for 1 h in buffer identical to that used for the primary antibody. After washing, bound antibody was visualised by a chromogenic reaction catalysed by the conjugated alkaline phosphatase using nitroblue tetrazolium and 5-bromo-4-chloro-3-indolyl phosphate as chromogenic substrate.

Controls included incubation without primary antibody and incubation with primary antibody which had been preincubated with the p50*met* protein which was used for immunization (1 μg peptide per 1 μl ascites fluid). Both these control experiments completely eliminated binding to proteins with $M_r$'s equivalent to those of c-*met* specific bands (data not shown). The c-*met* antibody and the corresponding blocking protein, p50*met*, was a generous gift of Dr Marianne Oskarsson, NCI-Frederick, USA. The available c-*met* specific antibody was not suitable for use in immunocytochemistry.

*Western blotting and immunocytochemistry of* c-kit *protein expression*

Western blotting was performed as described for c-*met* except that the buffer used for blocking and incubation was 100 mM Tris (pH 7.4) – 150 mM NaCl – 10% foetal calf serum – 2% BSA – 0.1% Triton X-100. The antibody was a mouse monoclonal (Boehringer Mannheim, Germany), and was used at a concentration of 10 μg $ml^{-1}$. A blocking peptide was not available for this antibody.

Immunocytochemistry was employed to determine the localisation of c-*kit* protein in the SCLC cells. Cell lines growing attached to the bottom of culture flasks were grown on 8-well slide glasses until attached, while cells growing in suspension culture were washed in phosphate buffered saline (PBS), placed on 8-well slide glasses in a small amount of PBS and allowed to dry completely. Cells on slide glasses were fixed in 1% formaldehyde in PBS for 10 min, washed in PBS and blocked with 10% foetal calf serum in PBS for

30 min. After washing in PBS, a polyclonal c-*kit* specific antibody was added at a concentration of 10 μg ml$^{-1}$ in PBS containing 4% BSA, and incubated for 4 h at room temperature. The primary antibody was raised in rabbits against a peptide with the sequence GSTASSSQPLLVHDDV, representing amino acids 961–976 in the C-terminal part of c-*kit* protein (Oncogene Science, Uniondale, NY). The specimens were washed in PBS containing 0.1% Tween-20 and incubated for 1 h with a FITC-conjugated swine anti-rabbit antibody (Dakopatts, Glostrup, Denmark) diluted 1:20 in PBS containing 4% BSA. An epifluorescence microscope (Aristoplan, Leica) equipped with filters appropriate for FITC fluorescence was used for viewing and photographing. Controls included incubation without the primary antibody and incubation with primary antibody which had been preincubated with the peptide used for immunisation (Oncogene Science). Preincubation was done with a 10-fold excess (by weight) of peptide.

### Results

The expression of mRNA transcripts of c-*met*, HGF/SF, c-*kit* and SCF in SCLC cell lines and xenografts is summarised in Table I. There was no systematical difference in expression pattern in the two model systems. In most cases there were similar expression levels in the two systems, but in some cases expression was slightly higher in a cell line than in the corresponding xenograft and vice versa.

Expression of c-*met* mRNA was detected in 22 of the 25 examined SCLC tumours (Figure 1). Most of the tumours expressed a single transcript with an electrophoretic mobility corresponding to a size of 7.5 kb. However, a few tumours (e.g. CPH-167 and DMS-456) also expressed a mRNA of about a 6 kb Figure 1), which was detectable only with the phosI probe (see Materails and methods).

The protein encoded by c-*met* was expressed in tumours expressing c-*met* mRNA (Figure 4), and in general the expression levels of c-*met* protein corresponded well with the levels of c-*met* mRNA. On the Western blots two bands with $M_r$'s of 145,000 (p145$^{MET}$, (Giordano *et al.*, 1989*b*)) and 170,000 (p170$^{MET}$) were observed in all positive tumours, the p145$^{MET}$ band being most prominent. In a few tumours (e.g. DMA-273 xeno and NCI-H69) the level of protein expression was relatively high despite the fact that c-*met* mRNA was quite low.

The level of c-*met* expression detected in different tumours varied widely (Figure 1). In general, tumours expressing c-*met* did so both when grown as cell lines and as nude mouse xenografts. However, a few tumours (e.g. CPH-54A, CPH-54B) expressed very low levels of c-*met* when grown *in vitro* as cell lines, whereas no expression was detectable in the corresponding xenografts.

Expression of c-*met* mRNA was also examined in normal adult human tissues, and high levels were detected in placenta and lung, while moderate expression was observed in heart, brain, liver, skeletal muscle and kidney (Figure 3). In pancreas, only trace levels of c-*met* expression was found.

In the examined SCLC tumours, expression of mRNA for the c-*met* ligand, HGF/SF, was detectable only in DMS-114 and NCI-N417 (Figure 1). Expression of HGF/SF was determined in various normal adult human tissues (MTN Blot, see Materials and methods), and expression was detected in heart, placenta, lung, liver, muscle (weak) and kidney (Figure 3).

Expression of c-*kit* mRNA was demonstrated in 22/25 SCLC cell lines (Figure 2). As was the case for c-*met*, the

**Table I** Expression of c-*met*, HGF/SF, c-*kit* and SCF in SCLC cell lines and nude mouse xenografts and in normal adult human tissues

| | *c-met* | | *HGF/SF* | | *c-kit* | | *SCF* | |
|---|---|---|---|---|---|---|---|---|
| *Tumour* | *line* | *xeno* | *line* | *xeno* | *line* | *xeno* | *line* | *xeno* |
| CPH-54A | (+) | – | – | – | – | – | – | – |
| CPH-54B | (+) | – | – | – | – | – | – | – |
| CPH-136A | NA | ++ | NA | – | NA | +++ | NA | + |
| CPH-136B | NA | + | NA | – | NA | +++ | NA | ++ |
| CPH-167 | NA | (+) | NA | – | NA | + | NA | ++ |
| CPH-186 | NA | ++ | NA | – | NA | + | NA | + |
| CPH-187 | NA | + | NA | – | NA | + | NA | + |
| DMS-53 | ++ | + | – | – | + | (+) | – | – |
| DMS-79 | +++ | +++ | – | – | +++ | +++ | + | + |
| DMS-92 | + | (+) | – | – | ++ | ++ | ++ | ++ |
| DMS-114 | – | – | ++ | ++ | – | – | (+) | (+) |
| DMS-153 | + | ++ | – | – | + | ++ | + | ++ |
| DMS-273 | ++ | + | – | – | (+) | – | (+) | – |
| DMS-406 | – | NA | – | NA | ++ | NA | ++ | NA |
| DMS-456 | ++ | ++ | – | – | + | + | – | – |
| GLC-2 | – | NA | – | NA | + | NA | – | NA |
| GLC-3 | + | + | – | – | +++ | +++ | – | – |
| GLC-14 | + | (+) | – | – | ++ | + | ++ | + |
| GLC-16 | + | (+) | – | – | +++ | ++ | + | + |
| GLC-19 | ++ | + | – | – | + | ++ | ++ | ++ |
| GLC-26 | + | NA | – | NA | ++ | NA | +++ | NA |
| GLC-28 | ++ | NA | – | NA | ++ | NA | +++ | NA |
| NCI-H69 | (+) | (+) | – | – | + | + | + | + |
| NCI-N417 | ++ | NA | + | NA | (+) | NA | (+) | NA |
| MAR-24H | ++ | NA | – | NA | + | NA | (+) | NA |
| Normal tissues | | | | | | | | |
| heart | + | | + | | + | | + | |
| brain | + | | – | | ++ | | + | |
| placenta | ++ | | +++ | | + | | + | |
| lung | ++ | | ++ | | +++ | | ++ | |
| liver | + | | ++ | | (+) | | (+) | |
| muscle | + | | (+) | | – | | + | |
| kidney | + | | + | | ++ | | ++ | |
| pancreas | – | | – | | – | | – | |

The level of expression was rated visually as none: '–', trace: '(+)', low: '+', intermediate '++', and high: '+++'. Xenografts marked 'NA' were not established as cell lines and vice versa.

**Figure 1** Northern blots demonstrating expression of the c-*met* proto-oncogene and of HGF/SF in SCLC cell lines **a**, and xenografts **b**. The two upper blots were probed with the phosI and the pmet5 probes, respectively. A 7.5 kb band was detected by both c-*met* probes, and an additional c-*met* specific band of approximately 6 kb (arrow) was seen in some tumours on blots probed with the phosI probe (top). The lower two blots demonstrate expression of a 6 kb HGF/SF transcript and of β-actin, respectively. An asterisk in **b**, marks non-specific hybridisation to the 4.8 kb ribosomal RNA band. Each lane contains 12 μg total RNA. The positions of the coelectrophoresed size markers or of the 18S (1.9 kb) and 28S (4.8 kb) ribosomal RNA bands are indicated.

**Figure 2** Northern blot demonstrating expression of the c-*kit* proto-oncogene and of SCF in SCLC cell lines **a**, and xenografts **b**. The upper panel was probed with a c-*kit* specific probe, the middle panel with a SCF specific probe, and the bottom panel with a β-actin specific probe. Each lane contains 12 μg total RNA. The positions of the 18S (1.9 kb) and 28S (4.8 kb) ribosomal RNA bands are indicated.

expression of c-*kit* seemed independent of the model system in which the tumours cells were grown. Coexpression of c-*met* and c-*kit* mRNA was found in 20 tumours.

Western blotting of c-*kit* protein demonstrated that there was very good correlation between the level of c-*kit* protein expression and c-*kit* mRNA expression (Figures 2 and 5). Two bands with $M_r$s and 145,000 and 120,000 were detected. The $M_r$ 145,000 band is the c-*kit* receptor while the $M_r$ 120,000 band is most likely a precursor (Blume-Jensen *et al.*, 1991).

Immunocytochemical analysis of cell lines with and without detectable expression of c-*kit* mRNA was also performed. The examined tumours were the c-*kit* mRNA positive DMS-92, DMS-153, DMS-273, DMS-406, GLC-14, GLC-16, GLC-19 and NCI-H69, and the c-*kit* mRNA negative CPH-54A, CPH-54B and DMS-114. In all tumours expressing c-*kit* mRNA, clear cell-membrane staining was observed together with some cytoplasmic staining with nuclear shadowing (Figure 6). In cell lines in which c-*kit* mRNA and protein were not detectable on Northern and Western blots (e.g. CPH-54A and CPH-54B), very weak membrane staining was detected. In all cases the staining could be completely eliminated by preincubation of the primary antibody with the peptide used for immunisation (Figure 6) or by omission of the primary antibody.

The ligand for c-*kit*, SCF, was expressed in 19/25 tumours, and coexpression of the c-*kit* receptor and its ligand was found in 18 of the examined tumours.

Normal adult human tissues frequently expressed both c-*kit* and SCF mRNA (Figure 3).

### Discussion

Twenty-two of 25 tumours (88%) expressed detectable amounts of c-*met* mRNA transcripts and c-*met* protein.

Two bands were detected by the 19S c-*met* monoclonal antibody on Western blots of proteins electrophoresed under reducing conditions. The smaller band represents the c-*met* β-chain ($p145^{MET}$) while the 170,000 band most likely represents uncleaved c-*met* precursor, $p170^{MET}$ (Giordano *et al.*, 1989*a*; Giordano *et al.*, 1989*b*). The α-chain with a $M_r$ of 50,000 ($p50^{MET}$), which is presumed to be derived from the amino-terminal part of the $p170^{MET}$ precursor (Tempest *et al.*, 1988), is likely not to be detected by the 19S antibody which was raised against a carboxyterminal c-*met*-protein (p50*met*).

It has been suggested that the size (9 kb) often reported for the c-*met* mRNA species expressed in various tissues (Park *et al.*, 1986; Park *et al.*, 1987; Prat *et al.*, 1991) is likely to be an overestimate (Park *et al.*, 1987). Examination of overlapping human c-*met* cDNA clones and heteroduplex analysis with full length mouse *met* cDNA, which is close to 7 kb long (Iyer *et al.*, 1990), indicated that the human c-*met* transcript is likely to be of approximately the same size (Park *et al.*, 1987). In accordance, the two c-*met* specific probes (pmet5 and phosI) used in the present study both detected a band with an electrophorectic mobility of 7.5 kb. One of the c-*met* probes used, pmet5, is known to recognise only one band on Northern blots (Park *et al.*, 1987). Examination of several Northern blots where an RNA size marker which included a band of 7.46 kb was coelectrophoresed, and of MTN Blots where the position of size marker bands are marked, repeatedly showed a c-*met* specific band with an electrophoretic mobility of 7.5 kb (Figures 1 and 3). To ascertain the authenticity of our pmet5 probe, we performed Southern blotting of *Taq*I digested human DNA and obtained the expected 3 bands (Dean *et al.*, 1987) of approximately 1.8, 3.2 and 11.0 kb (data not shown). Our results is further evidence that the actual size of the human c-*met* mRNA is close to 7.5 kb.

A few tumours expressed an additional c-*met* specific mRNA species with a size of approximately 6 kb detectable with the phosI probe but not with the pmet5 probe. In some tumours the phosI probe detects up to three different c-*met*

**Figure 3** Northern blots showing expression of c-*met* (top), HGF/SF (upper middle), c-*kit* (lower middle), and SCF (bottom) in normal adult human tissues (MTN Blot). Each lane contains 2 μg poly $A^+$ RNA. The positions of the coelectrophoresed size marker bands are indicated.

specific transcripts reported to be 6, 7, and 9 kb (Park *et al.*, 1987), and a 5 kb transcript of a fusion gene resulting from translocation of *tpr* (translocated promoter region) sequence from chromosome 1 to c-*met* sequence on chromosome 7 (Park *et al.*, 1986; Park *et al.*, 1987). As judged from its size, the 6 kb band is not likely to represent the 5 kb *tpr-met* fusion transcript, but rather one of the two other c-*met* transcripts of 6 and 7 kb. Thus, the examined SCLC tumours expressed only one additional c-*met* transcript when probed with the phosI probe, while other tumour cells have been reported to express at least two transcripts smaller than the 7.5 kb transcript detected by both c-*met* probes (Park *et al.*, 1986; Park *et al.*, 1987; Prat *et al.*, 1991).

If the c-*met* protein detected in the examined SCLC tumours encodes a functional receptor, this may be of importance for the behaviour of SCLC tumours in patients, since the c-*met* ligand, HGF/SF, is expressed in many normal tissues (Figure 3), and is present in the plasma (Nakamura *et al.*, 1989; Zarnegar *et al.*, 1990). We detected HGF/SF expression in several normal tissues (Figure 3), thus confirming that this growth factor is widely expressed (Rubin *et al.*, 1991; Higashio *et al.*, 1990; Yanagita *et al.*, 1992). However,

**Figure 4** Representative Western blots demonstrating c-*met* protein expression in SCLC xenografts (top) and cell lines (bottom). The positions of the coelectrophoresed size markers are indicated (in kD). The two c-*met* specific bands ($p145^{MET}$ and $p170^{MET}$) are indicated by arrows.

**Figure 5** Representative Western blots demonstrating c-*kit* protein expression in SCLC cell lines. The positions of the coelectrophoresed size markers are indicated (in kD). The two c-*kit* specific bands with $M_r$s of 145,000 and 120,000 are indicated by arrows.

among the examined SCLC tumours, only two expressed HGF/SF (Figure 1), and coexpression of c-*met* and HGF/SF was found in only one tumour (NCI-N417). Therefore the results do not indicate that an autocrine regulatory loop involving this receptor/ligand system is frequently active in SCLC.

Several normal tissues coexpressed c-*met* and its ligand HGF/SF, and c-*kit* and its ligand SCF (Figure 3). This suggests that these receptor-ligand systems may play a role in normal growth regulation in an autocrine or paracrine manner. It may be speculated that the expression of HGF/SF and SCF in many normal tissues could be of importance for the ability of SCLC tumour cells to metastasise to these tissues, or that HGF/SF or SCF produced in various organs might reach the lungs or sites with metastatic tumour spread and modulate the growth of c-*met* and c-*kit* positive SCLC tumours *in situ*. It is not known whether these ligands stimulate or inhibit SCLC growth; recent studies of various tumour cell lines, not including SCLC, have shown that HGF/SF may inhibit tumour cell growth (Tajima *et al.*, 1991; Shiota *et al.*, 1992), despite the fact that HGF/SF is a potent mitogen for hepatocytes (Nakamura *et al.*, 1989).

The fact that normal lung tissue expresses c-*met* mRNA does not necessarily imply that c-*met* is expressed in SCLC. The SCLC progenitor cell has not been identified with certainty, and it may represent only a minority of the cells present in normal lung tissue.

In one previous study (Prat *et al.*, 1991), three SCLC patient biopsies were examined for c-*met* protein expression by immunohistochemistry and none was detected. We detected c-*met* protein in the vast majority of cultured SCLC tumours. The cause of the apparent difference between our results and the results of Prat *et al.* (1991) is not clear at present, but it may be necessary to examine larger materials in order to determine whether there is an actual difference between c-*met* protein expression in SCLC patient biopsies and in cell lines.

The results obtained for the c-*kit* proto-oncogene confirm recent data demonstrating frequent expression of c-*kit* mRNA in SCLC (Sekido *et al.*, 1991). In our series 22/25 (88%) SCLC tumours expressed c-*kit* mRNA, which is in agreement with previous findings (Sekido *et al.*, 1991). Our

**Figure 6** Immunocytochemical demonstration of c-*kit* protein expression in representative SCLC cell lines. Micrographs on the left show results of incubation with c-*kit* antibody in a concentration of $10\,\mu g\,ml^{-1}$. Identically prepared samples incubated with primary antibody which had been pre-absorbed with blocking peptide are shown on the right. The cell lines shown are CPH-54A (top), DMS-153 (upper middle), GLC-16 (lower middle), and NCI-H69 (bottom). In all cases, micrographs of cells incubated with primary antibody which had or had not been preincubated with blocking peptide were exposed and reproduced under identical conditions. Magnification: × 1000.

results add important information to these previous findings by demonstrating that the c-*kit* mRNA is translated into protein. Immunocytochemical detection of very low levels of c-*kit* protein was possible in some of the cell lines in which c-*kit* mRNA or protein could not be demonstrated by Northern or Western blotting. This is most likely due to the very high sensitivity of immunocytochemical techniques. SCF mRNA was found to be expressed in a large proportion of SCLC, and coexpression of c-*kit* and its ligand SCF was demonstrated to be very frequent in SCLC, confirming very recent results (Hibi *et al.*, 1991).

Apparently, SCF is widely expressed in normal human tissues (Figure 3). The production of SCF by SCLC tumour cells and normal tissues may provide SCLC cells expressing the c-*kit* receptor with a growth advantage and may thus contribute to their malignant phenotype, provided that the receptor and its ligand are functional, and provided that a growth-stimulatory or an otherwise advantageous response is elicited in cells upon binding of SCF.

The proto-oncogenes c-*met* and c-*kit* can now be added to the long list of proto-oncogenes which are expressed in SCLC (Birrer & Minna, 1989; Mäkelä *et al.*, 1991). It could be speculated that expression of some of these genes may be a result of a general deregulation of transcription in cancer cells, leading to expression of genes that may not have any function in the tumour cells. However, there is evidence that expression of proto-oncogenes in SCLC is not the result of a non-specific general increase in transcription. For example, some proto-oncogenes, e.g. the c-*erb*B-2 gene is not expressed in the panel of tumours examined here (data not shown), and neither in another examined panel of SCLC (Schneider *et al.*, 1989). We also examined our SCLC tumour panel for expression of human serum albumin, which can be presumed to be of absolutely no importance for SCLC tumours, and found no detectable transcripts (data not shown). Indirectly, the type of data suggest that only genes which have a function in the tumour cells are expressed.

The fact that expresion of c-*met* and c-*kit* is found in

SCLC and in several normal tissues as reported by us and others (Prat *et al.*, 1991; Iyer *et al.*, 1990) suggests that these receptors may be involved in the regulation of cell behaviour in several tissues other than liver and stem cells. Thus, the designation HGFR and SCF receptor may not completely describe the function of these genes.

Recently, the ligands for c-*met* and c-*kit*, HGF/SF and SCF, have been cloned and expressed (Nakamura *et al.*, 1989; Miyazawa *et al.*, 1989; Zsebo *et al.*, 1990; Anderson *et al.*, 1990; Huang *et al.*, 1990), and it is possible to produce them in pure form. The availability of the ligands enables further studies of the function of the c-*met* and c-*kit* receptors in SCLC. It is of great potential interest that HGF/SF has been shown to modulate cell growth and motility (Nakamura *et al.*, 1989; Shiota *et al.*, 1992; Tajima *et al.*, 1991; Stoker, 1989; Gherardi *et al.*, 1989; Weidner *et al.*, 1990). HGF/SF is produced in normal lung tissue (Rubin *et al.*, 1991; Higashio *et al.*, 1990; Yanagita *et al.*, 1992) and thus may act on SCLC cells in the patient. We are currently investigating the possible effects of HGF/SF on SCLC cells.

The technical assistance of Ms Jette Røhrmann, Ms Jette Christiansen and Ms Kirsten Flod is greatly appreciated. We thank Professor Jørgen Rygaard, MD, The Bartholin Institute, for examining the immunofluorescence specimens, and the Danish Cancer Society, the Astrid Thaysen Foundation, the Gerda and Aage Haensch Foundation and Direktør Jacob Madsens og Hustru Olga Madsens Foundation for financial support.

**References**

ANDERSON, D.M., LYMAN, S.D., BAIRD, A., WIGNALL, J.M., EISENMAN, J., RAUCH, C., MARCH, C.J., BOSWELL, H.S., GIMPEL, S.D., COSMAN, D. & WILLIAMS, D.E. (1990). Molecular cloning of mast cell growth factor, a hematopoietin that is active in both membrane bound and soluble forms. *Cell*, **63**, 235–243.

BEPLER, G., JAQUES, G., NEUMANN, K., AUMÜLLER, G., GROPP, C. & HAVEMANN, K. (1987). Establishment, growth properties, and morphological characteristics of permanent human small cell lung cancer cell lines. *J. Cancer Res. Clin. Oncol.*, **113**, 31–40.

BERENDSEN, H.H., DE LEIJ, L., DE VRIES, E.G.E., MESANDER, G., MULDER, N.H., DE JONG, B., BUYS, C.H.C.M., POSTMUS, P.E., POPPEMA, S., SLUITER, H.J. & THE, H.T. (1988). Characterization of three small cell lung cancer cell lines established from one patient during longitudinal follow-up. *Cancer Res.*, **48**, 6891–6899.

BIRRER, M.J. & MINNA, J.D. (1989). Genetic changes in the pathogenesis of lung cancer. *Annu. Rev. Med.*, **40**, 305–317.

BLUME-JENSEN, P., CLAESSON-WELSH, L., SIEGBAHN, A., ZSEBO, K.M., WESTERMARK, B. & HELDIN, C.-H. (1991). Activation of the human c-*kit* product by ligand-induced dimerization mediates circular actin reorganization and chemotaxis. *EMBO J.*, **10**, 4121–4128.

BOTTARO, D.P., RUBIN, J.S., FALETTO, D.L., CHAN, A.M.-L., KMIECIK, T.E., VANDE WOUDE, G.F. & AARONSON, S.A. (1991). Identification of the hepatocytes growth factor receptor as the c-*met* proto-oncogene product. *Science*, **251**, 802–804.

CANTLEY, L.C., AUGER, K.R., CARPENTER, C., DUCKWORTH, B., GRAZIANI, A., KAPELLER, R. & SOLTOFF, S. (1991). Oncogenes and signal transduction. *Cell*, **64**, 281–302.

CARNEY, D.N., GAZDAR, A.F., BEPLER, G., GUCCION, J.G., MARANGOS, P.J., MOODY, T.W., ZWEIG, M.H. & MINNA, J.D. (1985). Establishment and identification of small cell lung cancer cell lines having classic and variant features. *Cancer Res.*, **45**, 2913–2923.

CHOMCZYNSKI, P. & SACCHI, N. (1987). Single-step method of RNA isolation by acid guanidinium thiocyanate-phenol-chloroform extraction. *Anal. Biochem.*, **162**, 156–159.

DE LEIJ, L., POSTMUS, P.E., BUYS, C.H.C.M., ELEMA, J.D., RAMAEKERS, F., POPPEMA, S., BROUWER, M., VAN DER VEEN, A.Y., MESANDER, G. & THE, T.H. (1985). Characterization of three new variant type cell lines derived from small cell carcinoma of the lung. *Cancer Res.*, **45**, 6024–6033.

DEAN, M., PARK, M., LE BEAU, M.M., ROBINS, T.S., DIAZ, M.O., ROWLEY, J.D., BLAIR, D.G. & VANDE WOUDE, G.F. (1985). The human *met* oncogene is related to the tyrosine kinase oncogenes. *Nature*, **318**, 385–388.

DEAN, M., O'CONNELL, P., LEPPERT, M., PARK, M., AMOS, J.A., PHILLIPS, D.G., WHITE, R. & VANDE WOUDE, G.F. (1987). Three additional DNA polymorphisms in the *met* gene and D7S8 locus: use in prenatal diagnosis of cystic fibrosis. *J. Pediatr.*, **111**, 490–495.

ENGELHOLM, S.A., VINDELØV, L.L., SPANG-THOMSEN, M., BRÜNNER, N., TOMMERUP, N., NIELSEN, M.H. & HANSEN, H.H. (1985). Genetic instability of cell lines derived from a single human small cell carcinoma of the lung. *Eur. J. Cancer Clin. Oncol.*, **21**, 815–824.

ENGELHOLM, S.A., SPANG-THOMSEN, M., VINDELØV, L.L., BRÜNNER, N., NIELSEN, M.H., HIRSCH, F., NIELSEN, A. & HANSEN, H.H. (1986). Comparison of characteristics of human small cell carcinoma of the lung in patients, *in vitro* and transplanted into nude mice. *Acta Pathol. Microbiol. Immunol. Scand. Sect. A.*, **94**, 325–336.

FEINBERG, A.P. & VOGELSTEIN, B. (1983). A technique for radiolabeling DNA restriction endonuclease fragments to high specific activity. *Anal. Biochem.*, **132**, 6–13.

GHERARDI, E., GRAY, J., STOKER, M., PERRYMAN, M. & FURLONG, R. (1989). Purification of scatter factor, a fibroblast-derived basic protein that modulates epithelial interactions and movement. *Proc. Natl Acad. Sci. USA*, **86**, 5844–5848.

GIORDANO, S., DI RENZO, M.F., NARSIMHAN, R.P., COOPER, C.S., ROSA, C. & COMOGLIO, P.M. (1989*a*). Biosynthesis of the protein encoded by the c-*met* proto-oncogene. *Oncogene*, **4**, 1383–1388.

GIORDANO, S., PONZETTO, C., DI RENZO, M.F., COOPER, C.S. & COMOGLIO, P.M. (1989*b*). Tyrosine kinase receptor indistinguishable from the c-*met* protein. *Nature*, **339**, 155–156.

GUNNING, P., PONTE, P., OKAYAMA, H., ENGEL, J., BLAU, H. & KEDES, L. (1983). Isolation and characterization of full-length cDNA clones for human α-, ,β- and γ-actin mRNAs: skeletal but not cytoplasmic actins have an amino-terminal cystein that is subsequently removed. *Mol. Cell Biol*, 787–795.

HIBI, K., TAKAHASHI, T., SEKIDO, Y., UEDA, R., HIDA, T., ARIYOSHI, Y. & TAKAGI, H. (1991). Coexpression of the stem cell factor and the c-*kit* genes in small-cell lung cancer. *Oncogene*, **6**, 2291–2296.

HIGASHIO, K., SHIMA, N., GOTO, M., ITAGAKI, Y., NAGAO, M., YASUDA, H. & MORINAGA, T. (1990). Identity of a tumor cytotoxic factor from human fibroblasts and hepatocyte growth factor. *Biochem. Biophys. Res. Commun.*, **170**, 397–404.

HUANG, E., NOCKA, K., BEIER, D.R., CHU, T.-Y., BUCK, J., LAHM, H.-W., WELLNER, D., LEDER, P. & BESMER, P. (1990). The hematopoietic growth factor KL is encoded by the Sl locus and is the ligand of the c-*kit* receptor, the gene product of the W locus. *Cell*, **63**, 225–233.

IYER, A., KMIECIK, T.E., PARK, M., DAAR, I., BLAIR, D., DUNN, K.J., SUTRAVE, P., IHLE, J.N., BODESCOT, M. & VANDE WOUDE, G.F. (1990). Structure, tissue-specific expression, and transforming activity of the mouse *met* protooncogene. *Cell Growth Differ.*, **1**, 87–95.

KYHSE-ANDERSEN, J. (1984). Electroblotting of multiple gels: a simple apparatus without buffer tank for rapid transfer of proteins from polyacrylamide to nitrocelluose. *J. Biochem. Biophys. Methods*, **10**, 203–209.

MÄKELÄ, T.P., MATTSON, K. & ALITALO, K. (1991). Tumour markers and oncogenes in lung cancer. *Eur. J. Cancer*, **27**, 1323–1327.

MIYAZAWA, K., TSUBOUCHI, H., NAKA, D., TAKAHASHI, K., OKIGAKI, M., ARAKAKI, N., NAKAYAMA, H., HIRONO, S., SAKIYAMA, O., GOHDA, E., DAIKUHARA, Y. & KITAMURA, N. (1989). Molecular cloning and sequence analysis of cDNA for human hepatocyte growth factor. *Biochem. Biophys. Res. Commun.*, **163**, 967–973.

NAKAMURA, T., NISHIZAWA, T., HAGIYA, M., SEKI, T., SHIMONISHI, M., SUGIMURA, A., TASHIRO, K. & SHIMIZU, S. (1989). Molecular cloning and expression of human hepatocyte growth factor. *Nature*, **342**, 440–443.

NAKAMURA, T. (1991). Structure and function of hepatocyte growth factor. *Prog. Growth Factor. Res.*, **3**, 67–86.

NALDINI, L., VIGNA, E., NARSIMHAN, R.P., GAUDINO, G., ZARNEGAR, R., MICHALOPOULOS, G.K. & COMOGLIO, P.M. (1991*a*). Hepatocyte growth factor (HGF) stimulates the tyrosine kinase activity of the receptor encoded by the proto-oncogene c-*MET* . *Oncogene*, **6**, 501–504.

NALDINI, L., WEIDNER, K.M., VIGNA, E., GAUDION, G., BARDELLI, A., PONZETTO, C., NARSIMHAN, R.P., HARTMANN, G., ZARNEGAR, R., MICHALOPOULOS, G.K., BIRCHMEIER, W. & COMOGLIO, P.M. (1991*b*). Scatter factor and hepatocyte growth factor are indistinguishable ligands for the *MET* receptor. *EMBO J.*, **10**, 2867–2878.

PARK, M., DEAN, M., COOPER, C.S., SCHMIDT, M., O'BRIEN, S.J., BLAIR, D.G. & VANDE WOUDE, G.F. (1986). Mechanism of *met* oncogene activation. *Cell*, **45**, 895–904.

PARK, M., DEAN, M., KAUL, K., BRAUN, M.J., GONDA, M.A. & VANDE WOUDE, G. (1987). Sequence of MET protooncogene cDNA has features characteristic of the tyrosine kinase family of growth-factor receptors. *Proc. Natl Acad. Sci. USA*, **84**, 6379–6383.

PETTENGILL, O.S., SORENSON, G.D., WURSTER-HILL, D., CURPHEY, T.J., NOLL, W.W., CATE, C.C. & MAURER, L.H. (1980). Isolation and growth characteristics of continous cell lines from small-cell carcinoma of the lung. *Cancer*, **45**, 906–918.

PRAT, M., NARSIMHAN, R.P., CREPALDI, T., NICOTRA, M.R., NATALI, P.G. & COMOGLIO, P.M. (1991). The receptor encoded by the human c-*MET* oncogene is expressed in hepatocytes, epithelial cells and solid tumors. *Int. J. Cancer*, **49**, 323–328.

RUBIN, J.S., CHAN, A.M.-L., BOTTARO, D.P., BURGESS, W.H, TAYLOR, W.G., CECH, A.C., HIRSCHFIELD, D.W., WONG, J., MIKI, T., FINCH, P.W. & AARONSON, S.A. (1991). A broad-spectrum human lung fibroblast-derived mitogen is a variant of hepatocyte growth factor. *Proc. Natl Acad. Sci. USA*, **88**, 415–419.

SAMBROOK, J., FRITSCH, E.F. & MANIATIS, T. (1989). *Molecular Cloning: A Laboratory Manual*, 2nd ed. Cold Spring Harbor Laboratory Press: New York.

SCHNEIDER, P.M., HUNG, M.C., CHIOCCA, S.M., MANNING, J. ZHAO, X.Y., FANG, K. & ROTH, J.A. (1989). Differential expression of the c-*erb*B-2 gene in human small cell and non-small cell lung cancer. *Cancer Res.*, **49**, 4968–4971.

SEKIDO, Y., OBATA, Y, UDEA, R., HIDA, T., SUYAMA, M., SHIMOKATA, K., ARIYOSHI, Y. & TAKAHASHI, T. (1991). Preferential expression of c-*kit* protooncogene transcripts in small cell lung cancer. *Cancer Res.*, **51**, 2416–2419.

SHIOTA, G., RHOADS, D.B., WANG, T.C., NAKAMURA, T. & SCHMIDT, E.V. (1992). Hepatocyte growth factor inhibits growth of hepatocellular carcinoma cells. *Proc. Natl Acad. Sci. USA*, **89**, 373–377.

SPANG-THOMSEN, M., NIELSEN, A. & VISFELDT, J. (1980). Growth curves of three human malignant tumors transplanted to nude mice. *Exp. Cell. Biol.*, **48**, 138–154.

STOKER, M. (1989). Effect of scatter factor on motility of epithelial cells and fibroblasts. *J. Cell. Physiol.*, **139**, 565–569.

TAJIMA, H,. MATSUMOTO, K. & NAKAMURA, T. (1991). Hepatocyte growth factor has potent anti-proliferative activity in various tumor cell lines. *FEBS Lett.*, **291**, 229–232.

TEMPEST, P.R., STRATTON, M.R. & COOPER, C.S. (1988). Structure of the *met* protein and variation of *met* protein kinase activity among human tumour cell lines. *Br. J. Cancer*, **58**, 3–7.

WEIDNER, K.M., BEHRENS, J., VANDEKERCKHOVE, J. & BIRCHMEIER, W. (1990). Scatter factor: molecular characteristics and effect on the invasiveness of epithelial cells. *J. Cell Biol.*, **111**, 2097–2108.

WEIDNER, K.M., ARAKAKI, N., HARTMANN, G., VANDEKERCKHOVE, J., WEINGART, S., RIEDER, H., FONATSCH, C., TSUBOUCHI, H., HISHIDA, T., DAIKUHARA, Y. & BIRCHMEIER, W. (1991). Evidence for the identity of human scatter factor and human hepatocyte growth factor., *Proc. Natl Acad. Sci. USA*, **88**, 7001–7005.

WILLIAMS, D.E., EISENMAN, J., BAIRD, A., RAUCH, C., VAN NESS, K., MARCH, C.J., PARK, L.S., MARTIN, U., MOCHIZUKI, D.Y., BOSWELL, H.S., BURGESS, G.S., COSMAN, D. & LYMAN, S.D. (1990). Identification of a ligand for the c-*kit* proto-oncogene. *Cell*, **63**, 167–174.

YANAGITA, K., NAGAIKE, M., ISHIBASHI, H., NIHO, Y., MATSUMOTO, K. & NAKAMURA, T. (1992). Lung may have an endocrine function producing hepatocyte growth factor in response to injury of distant organs. *Biochem. Biophys. Res. Commun.*, **182**, 802–809.

YARDEN, Y., KUANG, W.-J., YANG-FENG, T., COUSSENS, L., MUNEMITSU, S., DULL, T.J., CHEN, E., SCHLESSINGER, J., FRANCKE, U. & ULLRICH, A. (1987). Human proto-oncogene c-*kit*: a new cell surface receptor tyrosine kinase for an unidentified ligand. *EMBO J.*, **6**, 3341–3351.

ZARNEGAR, R., MUGA, S., RAHIJA, R. & MICHALOPOULOS, G. (1990). Tissue distribution of hepatopoietin-A: a heparin-binding polypeptide growth factor for hepatocytes. *Proc. Natl Acad. Sci. USA*, **87**, 1252–1256.

ZSEBO, K.M., WILLIAMS, D.A., GEISSLER, E.N., BROUDY, V.C., MARTIN, F.H., ATKINS, H.L., HSU, R.-Y., BIRKETT, N.C., OKINO, K.H., MURDOCK, D.C., JACOBSEN, F.W., LANGLEY, K.E., SMITH, K.A., TAKEISHI, T., CATTANACH, B.M., GALLI, S.J. & SUGGS, S.V. (1990). Stem cell factor in encoded at the Sl locus of the mouse and is the ligand for the c-*kit* tyrosine kinase receptor. *Cell*, **63**, 213–224.